# Scanning efficacy of p-Chips implanted in the wing and leg of the Big Brown Bat (*Eptesicus fuscus*)

**DOI:** 10.1093/jmammal/gyae030

**Published:** 2024-04-06

**Authors:** Shane D I Seheult, Raj Panchal, Alex V Borisenko, Patrick J Bennett, Paul A Faure

**Affiliations:** Department of Psychology, Neuroscience & Behaviour, McMaster University, Hamilton, ON L8S 4K1, Canada; Department of Psychology, Neuroscience & Behaviour, McMaster University, Hamilton, ON L8S 4K1, Canada; Department of Integrative Biology, University of Guelph, Guelph, ON N1G 2W1, Canada; Department of Psychology, Neuroscience & Behaviour, McMaster University, Hamilton, ON L8S 4K1, Canada; Department of Psychology, Neuroscience & Behaviour, McMaster University, Hamilton, ON L8S 4K1, Canada

**Keywords:** Chiroptera, handling time, marking, metacarpal, radio tag, scan time, tag visibility, tibia, Chiroptera, etiqueta de radio, marcaje, metacarpiano, tibia, tiempo de exploración, tiempo de manipulación, visibilidad de la etiqueta

## Abstract

Individual marking techniques are critical for studying animals, especially in the wild. Current marking methods for bats (Order Chiroptera) have practical limitations and some can cause morbidity. We tested the p-Chip (p-Chip Corp.)—a miniaturized, laser light-activated microtransponder—as a prospective marking technique in a captive research colony of Big Brown Bats (*Eptesicus fuscus*). We assessed long-term readability and postimplantation effects of p-Chips injected subcutaneously above the second metacarpal (wing; *n* = 30) and the tibia (leg; *n* = 13 in both locations). Following implantation (Day 0), p-Chips were scanned with a hand-held ID reader (wand) on postimplantation days (PIDs) 1, 8, 15, 22, 32, 60, 74, 81, 88, 95, and over 1 year later (PID 464). For each trial, we recorded: (1) animal handling time; (2) scan time; (3) number of wand flashes; (4) p-Chip visibility; and (5) overall condition of the bat. Average scan times for p-Chips implanted in both the wing and leg increased over the duration of the study; however, the number of wand flashes decreased, suggesting that efficacy of p-Chip recording increased with user experience. Importantly, over 464 days both the visibility and readability of p-Chips in the wing remained high and superior to tags in the leg, establishing the second metacarpal as the preferred implantation site. Observed morbidity and mortality in captive bats with p-Chips was similar to baseline values for bats without these tags. Because scan efficiency on PID 464 was comparable with earlier days, this indicates that p-Chips implanted in the wing may be suitable as a long-term marking method. Our provisional results suggest that p-Chips are viable for extended field testing to see if they are suitable as an effective alternative to traditional methods to mark bats.

## Teaser

We assessed the p-Chip technology as a viable marking technique in bats by measuring the time for handling bats and scanning tags implanted in the wing and leg. We found that the wing was the preferred site. The p-Chip technology may be an effective marking tool for bats.

## Resumen

Las técnicas de marcaje individual son fundamentales para el estudio de los animales, especialmente en la naturaleza. Los métodos actuales de marcaje de murciélagos (Chiroptera) tienen limitaciones prácticas y algunos pueden causar morbilidad. Probamos el p-Chip (p-Chip Corp.)—un microtranspondedor miniaturizado activado por luz láser—como técnica de marcaje prospectivo en una colonia en cautiverio de murciélagos morenos (*Eptesicus fuscus*). Se evaluó la legibilidad a largo plazo y los efectos pos-implantación de los p-Chips inyectados subcutáneamente sobre el segundo metacarpiano (ala; *n* = 30) y la tibia (pata; *n* = 13 en ambas localizaciones). Tras la implantación (día 0), se escanearon los p-Chips con un lector de identificación manual (vara) en los días posteriores a la inyección (PID) 1, 8, 15, 22, 32, 60, 74, 81, 88, 95, y más de un año después (PID 464). En cada ensayo se registró: (1) el tiempo total de manipulación del animal; (2) el tiempo de exploración; (3) el número de destellos de proximidad del lector; (4) la visibilidad del p-Chip; y (5) el estado general del murciélago. Los promedios del tiempo de escaneado de los p-Chips implantados tanto en el ala como en la pata aumentaron a lo largo del estudio; sin embargo, el número de destellos del lector disminuyó, lo que sugiere que la eficacia del registro del p-Chip aumentó con la experiencia del usuario. A lo largo de 464 días, tanto la visibilidad como la legibilidad de los p-Chips en el ala siguieron siendo altas y superiores a las de las etiquetas en la pata, lo que estableció el segundo metacarpiano como el lugar preferido de implantación. La morbilidad y mortalidad observadas en murciélagos en cautiverio con p-Chips fue similar a los valores de referencia de los murciélagos sin estas marcas. Dado que la eficacia del escaneado en el PID 464 fue comparable a la de días anteriores, es probable que los p-Chips implantados en el ala sean adecuados como método de marcado a largo plazo. Nuestros resultados provisionales sugieren que los p-Chips son viables para pruebas de campo prolongadas como alternativa prospectiva a los métodos tradicionales de marcaje de murciélagos.

Animal identification with individual marking techniques is important for addressing many questions about wildlife biology. The efficacy of a marking technique depends on the likelihood of follow-up encounters with tagged individuals, the permanence of the mark, the ease of mark recognition, and minimization of its impact on animal health, well-being, and behavior (Buchler 1976; Kunz and Weise 2009; Silvy et al. 2012). Thus, animal marking involves balancing performance criteria with ethical considerations (Powell and Proulx 2003). Among mammals, bats (Order Chiroptera) have been a popular subject for mark–recapture studies, leading to important insights into their homing abilities (Mohr 1934; Trapido and Crowe 1946; Cockrum 1956; Dwyer 1966; O’Donnell 2001; Fleming and Eby 2003; Gibbons and Andrews 2004; Campbell et al. 2006; Chaveri et al. 2007; Goldshtein et al. 2021), population dynamics (Dwyer 1969; Humphrey 1971), growth rate (Gibbons and Andrews 2004), survivorship (Leigh and Handley 1991; Hoyle et al. 2001; Young 2001; O’Donnell 2002), development (Kunz and Stern 1995; Kunz and Hood 2000), and behavior (Dwyer 1970; Bradbury 1977; McCracken and Wilkinson 2000; Reeder et al. 2006; Zubaid et al. 2006).

Many techniques have been developed for marking bats in the field and laboratory (Kunz and Weise 2009); however, the applicability and impact of a particular technique may differ between species and even individuals, often depending on the ecological and life history context (Bonaccorso et al. 1976; Kunz and Weise 2009; Silvy et al. 2012). Forearm bands are the most widely and continuously used bat marking technique (Trapido and Crowe 1946; Hitchcock 1965; Greenhall and Paradiso 1968; Stebbings 1978; Phillips 1985). Due to its early adoption, relatively low cost, and ease of application, banding has resulted in the largest and most comprehensive global data sets on bat longevity and movements compared to other marking techniques. Banding efficacy and impact on bat health depend on the species, situation, and type of band (Bonaccorso et al. 1976; Vardon and Tidemann 2000). In some cases, bands may cause injury, decrease foraging success, and increase morbidity and mortality (Herried and Davis 1960; Perry and Beckett 1966; Rybar 1973; Pierson and Fellers 1993; Norman et al. 1999; Baker et al. 2001; O’Shea et al. 2004; Dietz et al. 2006). Such adverse effects have inspired a continued search for alternative marking approaches.

Radio frequency identification (RFID) markers, specifically passive integrative transponder (PIT) tags, are commonly used to mark bats (Barnard 1989; Want 2006; Voulodimos et al. 2010). Subcutaneous PIT tags are implanted via needle injection, typically along the back between the shoulder blades (Banard 1989; Rigby et al. 2012). Each PIT tag transmits a unique radio frequency serial identification (ID) number when its solenoid antenna receives radio wave energy from an associated reader (Want 2006). The use of PIT tags also has trade-offs (Barclay and Bell 1988; Rigby et al. 2012). The PIT tag injection may stress the animal because it is invasive (and potentially dangerous) and requires a large (e.g., 12-gauge) needle. Injected PIT tags are not visible to the naked eye but can be felt by palpating the injection site, further increasing animal handling. PIT tags can also move under the skin after implantation (Banard 1989) and possibly be expelled from the body through the implantation site (Kunz and Weise 2009). Several studies in bats have examined the impact of PIT tags on recapture rates, body mass, body condition, and reproductive success, and found no differences between tagged and untagged animals (Murray and Fuller 2000; Neubaum et al. 2005; Rigby et al. 2012). The placement of PIT tag reader arrays in cave entrances has been shown to have minimal impacts on bat flight and behavior (Britzke et al. 2014).

More recently, a miniaturized alternative to PIT tags has been developed: the p-Chip (p-Chip Corp., Chicago, Illinois; https://p-chip.com). The p-Chip is a flat square 500 × 500 µm microtransponder semiconductor tag (mass ~85 µg) activated by red laser light emitted by a compatible hand-held ID reader wand connected to a computer via a universal serial bus (USB) cable. The wand continuously emits lower-power laser light when it is idle; however, as the beam approaches and illuminates the photosensitive cells on the top surface of the p-Chip, the laser operates in higher-power pulsed burst mode and the beam flashes (i.e., flickers) in intensity (Gruda et al. 2010; PharmaSeq. 2012). When activated, the p-Chip transmits a unique 9-digit serial ID number as a radio signal that is detected by the sensor of the wand. This ID number is then transmitted to the computer and recorded by p-Chip Reader software. The ID readout is nearly instantaneous (<0.01 s).

For a successful read, the p-Chip must be in close proximity to the light-emitting tip of the wand and have its photocells facing the wand, with no opaque materials in between. For this reason, p-Chips are often surface-mounted on objects (Jolley-Rogers et al. 2012; Mandecki et al. 2017) or animals (Robinson et al. 2009; Robinson and Mandecki 2011; Tenczar et al. 2014; Mandecki et al. 2016; Hamilton et al. 2019). When used subcutaneously, p-Chips are injected in areas where the skin is thin, translucent, and hairless (Gruda et al. 2010; Chen et al. 2013; Delcourt et al. 2018). Due to their polymer coating, p-Chips are resilient to chemicals, high temperatures, repeated freezing/thawing, and placement in liquid nitrogen (PharmaSeq. 2012). Therefore, once implanted, p-Chips are expected to function indefinitely.

To date, p-Chip technology has been successfully adopted for tagging honeybees (Tenczar et al. 2014), ants (Robinson et al. 2009, 2014), and fish (Chen et al. 2013; Delcourt et al. 2018; Faggion et al. 2020; Moore and Brewer 2021). Among mammals, the only published protocol is for laboratory mice with transponders implanted subcutaneously in the pinna or near the base of the tail, with the latter identified as the preferred location (Gruda et al. 2010). A conference abstract reports using p-Chips to mark bats in the field, but without details of the implantation technique or tag placement (Ngamprasertwong et al. 2022). Our goal was to evaluate the p-Chip as a prospective method to mark bats. We did this by testing the hypothesis that there was no difference in scanning efficiency over time for p-Chips implanted subcutaneously in 2 anatomical locations—the second metacarpal (i.e., the wing) and the tibia (i.e., the leg)—using a captive research colony of Big Brown Bats (*Eptesicus fuscus*).

## Materials and methods

### Animals.

Thirty Big Brown Bats (*E*. *fuscus*) were used in this study. All bats were either wild-caught as adults in southern Ontario (*n* = 9) or direct descendants born in captivity (*n* = 21). Bats were housed in a husbandry facility at McMaster University where temperature and light varied seasonally following ambient conditions (Skrinyer et al. 2017). The facility consisted of 2 indoor enclosures (2.5 × 1.5 × 2.3 m; l × w × h), 1 of which was connected through a hole in the wall to a larger outdoor flight area (2.5 × 3.8 × 2.7 m) that bats could freely access. Food (mealworms; *Tenebrio molitor*) and water were provided ad libitum. For the bats we studied (*n* = 30), the mean ± standard deviation (SD) mass was 18.7 ± 4.2 g (range: 11.6 to 30.8 g) and forearm length was 45.25 ± 1.64 mm (range: 40.50 to 47.95 mm). Each bat was individually identified with a colored, numbered, plastic split-ring forearm band and a PIT tag injected subcutaneously between the shoulder blades. Bats were monitored for health changes throughout the study. All experimental procedures were approved by the Animal Research Ethics Board of McMaster University and conformed to the *Guide to the Care and Use of Experimental Animals* published by the Canadian Council of Animal Care and the ASM guidelines for research on live animals (Sikes et al. 2016).

### Tag implantation.

**Fig. 1.**
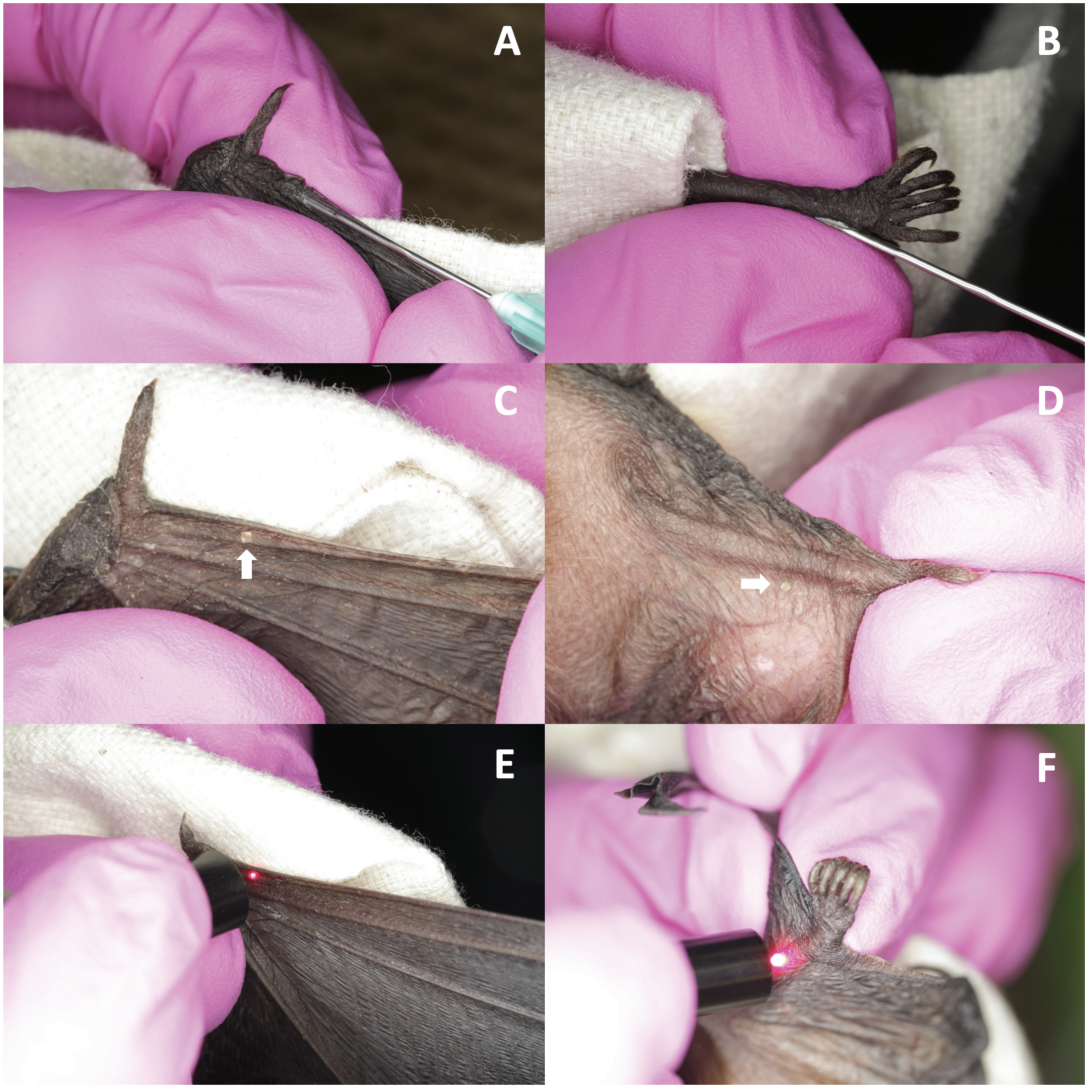
Subcutaneous implantation and laser scanning of p-Chips in the wing and leg of the Big Brown Bat. (A) Injection of p-Chip parallel to the second metacarpal. (B) Injection of p-Chip near the base of the foot parallel to the tibia. (C) Visibility of p-Chip against the second metacarpal and (D) in the tissue beside the tibia (location of the p-Chip in both images is indicated by a *white arrow*). (E) Using the wand to illuminate (scan) p-Chips implanted in the wing and (F) the leg. p-Chip dimension = 500 × 500 µm.

p-Chips were injected subcutaneously in hand-restrained bats by the same operator (AVB) on 11 November 2019, using preloaded, sterile, flat-tipped 21-gauge needles with plunger purchased from p-Chip Corp., in 2 predefined locations (Fig. 1): (1) wing (primary site; Fig. 1A and C; *n* = 30)—dorsally over the proximal part and parallel to the right second metacarpal, approximately 1 cm from the proximal carpal joint; and (2) leg (secondary site; Fig. 1B and D; *n* = 13)—parallel to the midpoint of the right tibia along its dorsal side.

Important considerations in site selection were accessibility for implantation and later scanning with the wand, transparency of the skin for tag visibility, and minimizing risk of damaging blood vessels, nerves, or tendons during injection. Implanted p-Chips were positioned with their photocells facing outward (i.e., away from the bone and toward the exterior skin surface). Hemostatic powder and/or small ephrin balls were used to stop any bleeding observed at the injection site. Following injection, p-Chips were scanned with the laser reader wand (model WA-4000) and the data were automatically transferred into a Microsoft Excel spreadsheet using p-Chip Reader software provided by the manufacturer (Fig. 1E and F). Of 30 bats tested, 13 were tagged in both sites and the remainder were tagged in the wing only (Table 1).

**Table 1.** Post-implantation day (PID) recording dates for p-Chips implanted in the wing and leg of the Big Brown Bat, *Eptesicus fuscus*

PID #	Date (YYYY-MM-DD)	# Bats (wing only)	# Bats (wing + leg)	Total # bats
0^a^	2019-11-11	17	13	30
1	2019-11-12	17	13	30
8	2019-11-19	17	13	30
15	2019-11-26	17	13	30
22	2019-12-03	16	13	29
32	2019-12-13	16	13	29
60	2020-01-10	15	13	28
74	2020-01-24	15	13	28
81	2020-01-31	15	13	28
88	2020-02-07	15	13	28
95	2020-02-14	15	13	28
464	2021-02-17	5	5	10

^a^PID 0 = day of p-Chip implantation.

### p-Chip scanning.

Two persons (SDIS, RP) conducted each scanning session. The first person, the “handler,” restrained and manipulated the bat and positioned the wand to be in close proximity to the p-Chip for a successful read. The second person, the “recorder,” operated the digital timer, software, and recorded data. The roles of the 2 individuals were randomized at the start of each session and were switched when approximately half of the bats had been recorded. After scanning, bats were returned to the husbandry facility where they remained until the next session. A movie illustrating the procedure of p-Chip implantation and scanning in the wing of *E. fuscus* is available (Supplementary Data SD1).

We quantified p-Chip readability separately for each implantation site by recording the time spent locating and scanning tags. After the handler removed a bat from its cage, the recorder started a digital timer to mark the start of handling time, defined as the duration (s) between the initial restraining of the bat and the end of the scanning trial. Working quickly, the handler manipulated and oriented the bat so that its p-Chip implantation site in the wing or leg was accessible for scanning. At this point, the visibility of the p-Chip was assessed by the handler using a yes/no nominal scale. Once the handler picked up the wand, the recorder started a second (lap) timer to measure the p-Chip scan time for that location. The handler then directed the laser beam of the wand back and forth over the p-Chip to obtain a read.

When the laser is in close proximity to the p-Chip, the light intensity briefly increases to activate photocells of the transponder (PharmaSeq. 2012). In practice, these proximity “wand flashes” helped us to obtain a successful read. When a unique 9-digit ID number from the transponder was detected by the p-Chip Reader software, it was automatically logged to an Excel spreadsheet and an audible tone was emitted from the computer. Following a successful read, the recorder stopped the timers. Conversely, if the read was unsuccessful, no audible tone was produced and the handler would continue scanning the implantation site. If a p-Chip was not read within 45 s of handling time, the handler proceeded with a 2-min free scan and directed the laser beam both dorsally and ventrally, on and away from the original implantation site, as a last attempt to read a chip that may have shifted laterally (i.e., translocated) and/or reoriented and flipped in situ so that its photocells no longer faced outward. When a p-Chip was not read within 2 min 45 s, the tag was recorded as unreadable for that session. For bats with p-Chips in both the wing and leg, a coin flip determined which location to scan first and the bat was returned to its cage before repeating the above procedure for the other site.

We scanned bats routinely from November 2019 to February 2020, except between 13 December 2019 and 10 January 2020 (Table 1). Owing to the COVID-19 pandemic, no data were collected from February 2020 until February 2021 when a subsequent recording session was conducted on PID 464, approximately 1 year later. For each trial, we recorded: (1) handling time (s); (2) scan time (s); (3) number of wand flashes (a proxy for scan attempts); (4) p-Chip visibility (yes/no); and (5) comments on overall condition of the bat. Note that we did not record handling time for the tibia on PID 0 and p-Chip visibility was recorded starting on PID 22.

### Data analyses.

**Fig. 2.**
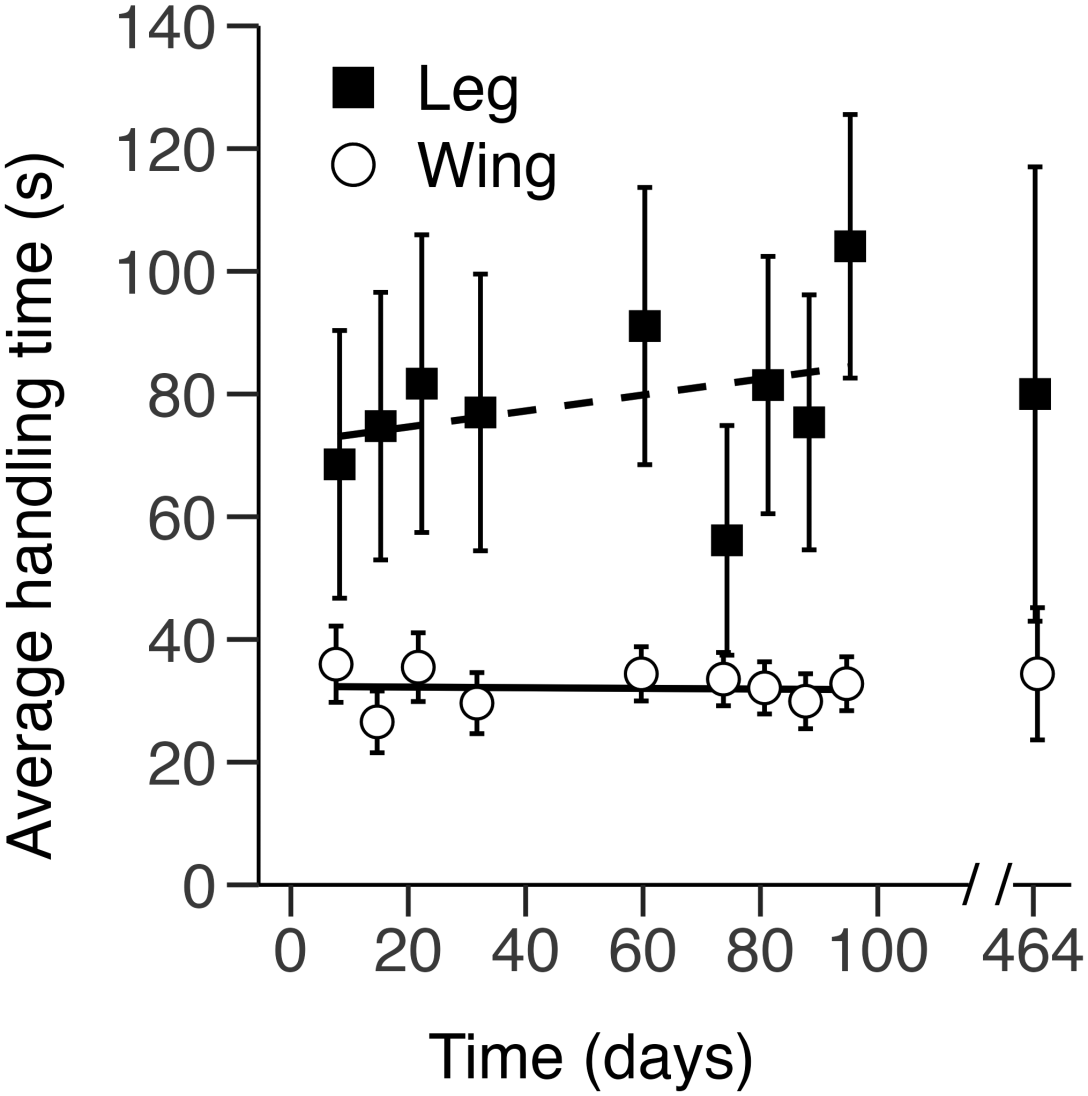
Bat handling times per recording day for p-Chips implanted in the wing and leg. Mean ± SE handling times were measured separately for p-Chips implanted in the second metacarpal (wing, *n* = 30) and tibia (leg, *n* = 13) from PID 8 to PID 95, with a subsequent recording session ~1 year later on PID 464. *Dotted* and *dashed lines* represent the best-fitting, mixed-model regression lines for p-Chips implanted in the wing (*open circles*) and leg (*closed squares*). For data points collected on the same day, the markers have been displaced ±0.3 along the *x-axis* for clarity.

**Fig. 3.**
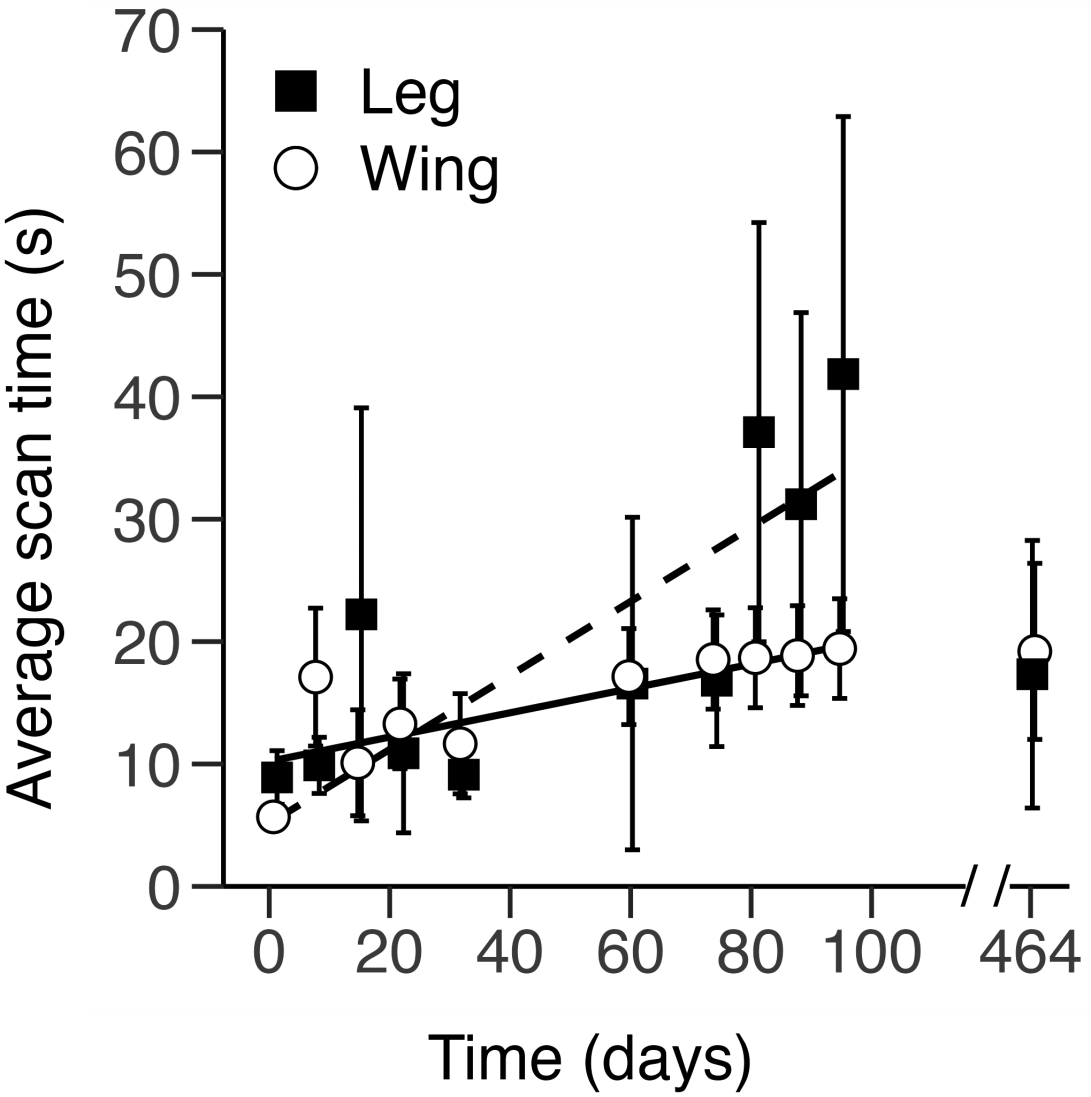
Scan times per recording day for p-Chips implanted in the wing and leg. Mean ± SE scan times were recorded separately for p-Chips implanted in the second metacarpal (wing, *n* = 30) and tibia (leg, *n* = 13) from PID 1 to PID 95, with a subsequent recording session ~1 year later on PID 464. Data do not include occurrences of unsuccessful p-Chip reads when the maximum scan time was reached (165 s). *Dotted* and *dashed lines* represent the best-fitting, mixed-model regression lines for p-Chips implanted in the wing (*open circles*) and leg (*closed squares*). For data points collected on the same day, the markers have been displaced ±0.3 along the *x-axis* for clarity.

**Fig. 4.**
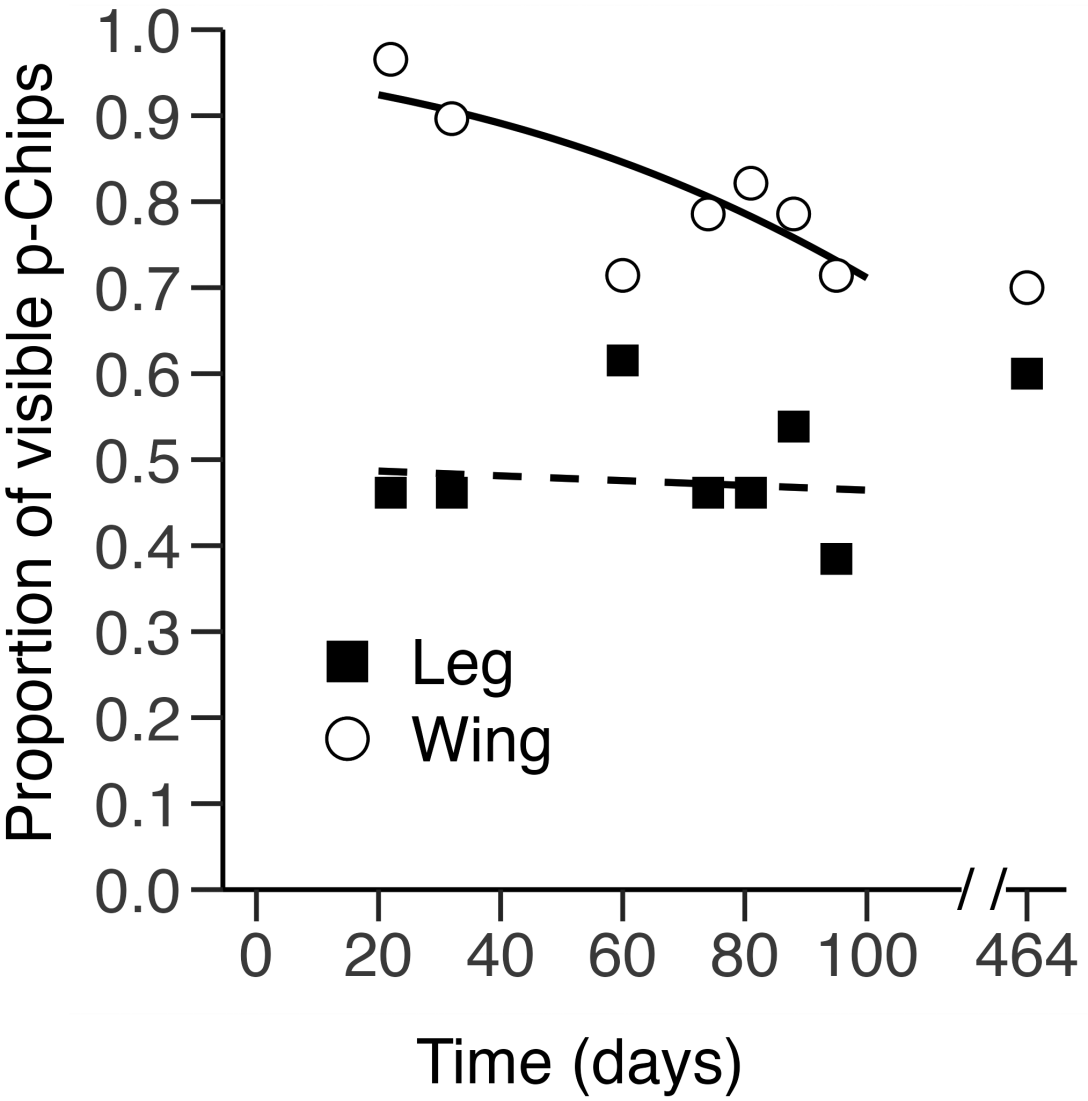
Tag visibility per recording day for p-Chips implanted in the wing and leg. Data illustrate the proportion of p-Chips implanted in the second metacarpal (wing, *n* = 30) and tibia (leg, *n* = 13) that were visible to the naked eye from PID 22 to PID 95, with a subsequent recording session ~1 year later on PID 464. Visibility measured according to the hander’s subjective judgement using a nominal yes/no scale. *Dotted* and *dashed lines* represent the best-fitting, mixed-model regression lines for p-Chips implanted in the wing (*open circles*) and leg (*closed squares*).

**Fig. 5.**
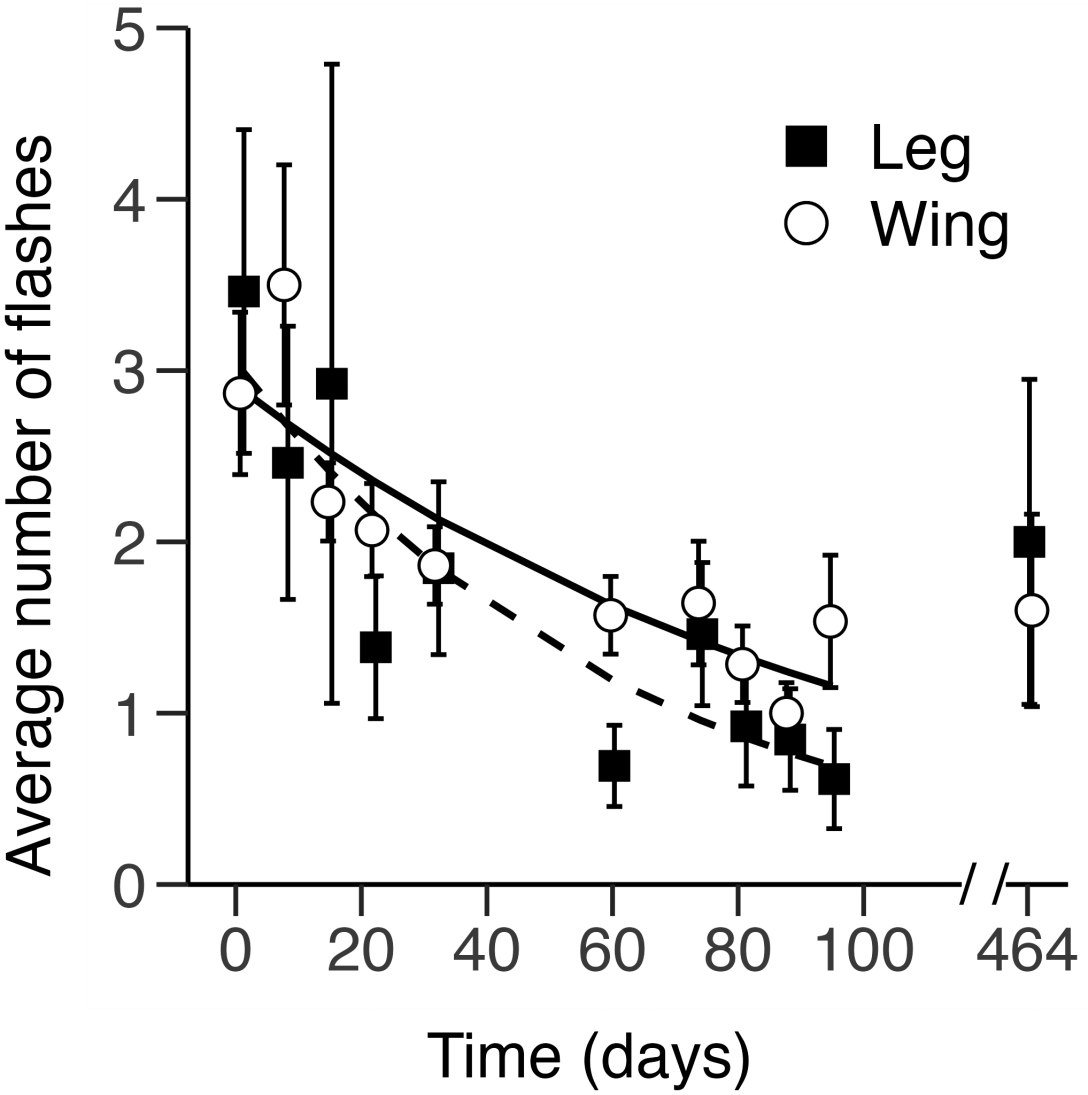
Wand flashes per recording day for p-Chips implanted in the wing and leg. Shown are the mean ± SE number of wand flashes recorded in p-Chips implanted in the second metacarpal (wing, *n* = 30) and tibia (leg, *n* = 13), prior to a successful p-Chip read from PID 1 to PID 95, with a subsequent recording session ~1 year later on PID 464. *Dotted* and *dashed lines* represent the best-fitting, mixed-model regression lines for p-Chips implanted in the wing (*open circles*) and leg (*closed squares*). For data points collected on the same day, the markers have been displaced ±0.3 along the *x-axis* for clarity.

**Fig. 6.**
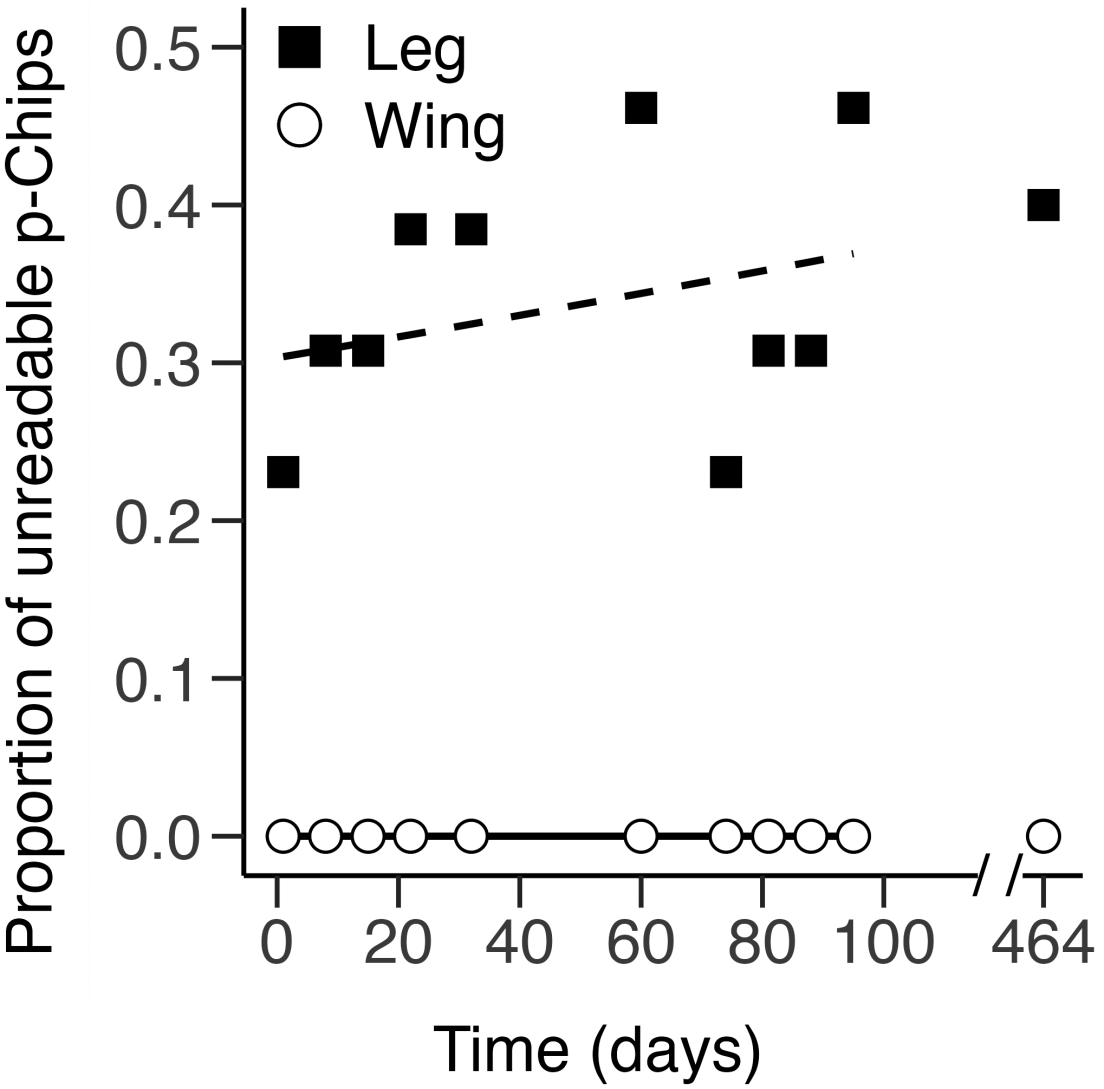
Unreadable tags per recording day for p-Chips implanted in the wing and leg. Shown are the proportion of unreadable p-Chips in the second metacarpal (wing, *n* = 30) and tibia (leg, *n* = 13) over the duration of the study. *Dotted* and *dashed lines* represent the best-fitting, mixed-model regression lines for p-Chips implanted in the wing (*open circles*) and leg (*closed squares*).

Data analysis was conducted in R (R Core Team 2021) and visualized with the *ggplot2* (Wickham 2016), *plotrix* (Lemon 2006), and *ggbreak* (Xu et al. 2021) packages. Unless stated otherwise, summary data are displayed as the mean ± standard error (SE), with applicable measures reported with 95% confidence intervals (CIs). Pearson’s product-moment correlation (*r*) evaluated the relationship between handling time and scan time. Two-sample *t*-tests were used to compare handling and scan times between handlers. Dependent variables were evaluated quantitatively with generalized linear mixed-effects models (GLMMs) that included tag Location (leg vs. wing) and Day as fixed effects and an intercept for each bat as a random effect. Specifically, the models for handling time and scan time were fit to the data using the *lmer* function, whereas p-Chip visibility, number of wand flashes, and the proportion of unreadable p-Chips were modeled using the *glmer* function in the *lme4* and *lmerTest* packages (Bates et al. 2015; Kunznestova et al. 2017). We excluded the PID 464 data to ensure the GLMM analyses were not skewed by an extreme value. The main effects of Day and Location, and Day × Location interactions for handling time and scan time were evaluated with *F*-tests using degrees of freedom calculated with Satterthwaite’s method (Satterthwaite 1946). By contrast, the fixed effects for p-Chip visibility, wand flashes, and unreadable p-Chips were evaluated with Chi-square (χ^2^) tests computed by the *Anova* function in the *car* package (Fox and Weisberg 2019). The GLMMs for the binomial variables p-Chip visibility and unreadable p-Chips were fit using the *logit* link-function, whereas the model for the continuous variable number of wand flashes was fit using a Poisson regression with a *log* link-function. The models for every variable fit the data reasonably well and we show best-fitting curves for each Location and variable (Figs. 2–6).

## Results

To evaluate scanning efficacy, we compared bat handling times (Fig. 2) and p-Chip scanning times (Fig. 3) for the wing and leg implantation sites. By definition, handling time was always larger than the respective scan time, and the 2 paired measures were strongly positively correlated (*r* = 0.916, *t*_388_ = 44.85, *P* < 0.001, 95% CI [0.90, 0.93]).

### Handling time.

Handling times were, on average, longer and more variable when recording p-Chips in the leg versus the wing (Fig. 2). The distribution of handling times contained outliers and was positively skewed (range = [3, 255], median = 22, mean = 47.1); hence, we analyzed log-transformed data. The main effect of Location was significant (*F*_1,369_ = 19.1, *P* < 0.001), but the main effect of Day (*F*_1,346_ = 0.26, *P* = 0.61) and the Location × Day interaction (*F*_1,346_ = 0.38, *P* = 0.54) were not. Similar results were obtained when we analyzed nontransformed handling time. In summary, handling time was significantly longer when recording p-Chips in the leg versus the wing, and this finding did not vary over the course of the study.

We also compared handling times between the 2 bat handlers. The average handling time to record p-Chips implanted in the wing was 39 and 26 s for the 2 handlers, and this difference was significant (*t*_244.36_ = 4.36, *P* < 0.001, 95% CI [8, 20]). The mean handling time to record p-Chips implanted in the leg was 83 and 75 s for each handler, but this difference was not significant (*t*_106.63_ = 0.57, *P* = 0.573, 95% CI [−20, 37]).

### Scan time.

Scan times were less variable for p-Chips implanted in the wing versus the leg (Fig. 3). Similar to handling time, the distribution of data for scan time contained outliers and was positively skewed (range = [1, 157], median = 5, mean = 16.3); thus, we analyzed log-transformed data. There was no main effect of Location (*F*_1,367_ = 0.86, *P* = 0.35); hence, scan times for p-Chips implanted in the wing and leg were similar (Fig. 3). However, the main effect of Day (*F*_1,342_ = 15.1, *P* < 0.001) was significant; average scan times increased between PID 1 and PID 95 for p-Chips implanted in both the wing and leg. The increase in scan time across days was slightly greater for p-Chips located in the leg (0.8% per day) compared to the wing (0.5% per day), but the Location × Day interaction was not significant (*F*_1,342_ = 0.76, *P* = 0.38). An analysis of nontransformed scan time data yielded similar results, except that analysis also found a significant Location × Day interaction (*F*_1,343_ = 6.27, *P* < 0.013). Unlike the result for handling time, our analysis failed to find a difference in scan time for p-Chips implanted in the wing and leg. Instead, we found evidence for a small but significant increase in scan time from PID 1 to PID 95 that may be slightly greater for p-Chips implanted in the leg. There was no difference in scan times between the 2 bat handlers for p-Chips located in the wing (*t*_240.67_ = 0.71, *P* = 0.479, 95% CI [−3, 7]) and leg (*t*_65.55_ = −0.66, *P* = 0.512, 95% CI [−23, 12]).

### p-Chip visibility.

Compared to the skin of the leg, the bat wing membrane is thinner, less opaque, and sits tightly on the digits; hence, there is less room for p-Chips to become displaced or flip at the implantation site. For these reasons, we expected p-Chips to remain more visible in the wing than in the leg. The visibility of p-Chips in the wing was initially close to 100% and only decreased to ~70% between PID 22 and PID 95 (Fig. 4). In contrast, less than half of the p-Chips implanted in the leg were visible on PID 22, a percentage that remained relatively constant over time (Fig. 4).

The main effect of tag Location was significant (χ^2^ = 26.78, df = 1, *P <* 0.001); visibility was greater for p-Chips implanted in the wing compared to in the leg (Fig. 4). There was no main effect of Day (χ^2^ = 3.44, df = 1, *P =* 0.064) and the Location × Day interaction (χ^2^ = 2.75, df = 1, *P =* 0.097) was also nonsignificant. Given the trends in our data (Fig. 4), the failure to find a Location × Day interaction was surprising. We therefore decided to examine the effect of Day separately for each Location and found a significant effect for p-Chips implanted in the wing (χ^2^ = 6.09, df = 1, *P =* 0.014) but not in the leg (χ^2^ = 0.02, df = 1, *P =* 0.88).

### Wand flashes.

The average number of wand flashes decreased by ~47% in the wing and ~82% in the leg between PID 1 and PID 95 (Fig. 5). The main effects of Location (χ^2^ = 6.02, df = 1, *P =* 0.014) and Day (χ^2^ = 99.32, df = 1, *P <* 0.001) were significant. The Location × Day interaction (χ^2^ = 4.83, df = 1, *P =* 0.023) was also significant, with the effect of day being smaller for p-Chips implanted in the leg. A follow-up analysis examining the effect of Day separately for each Location found a significant effect of Day for p-Chips implanted in both the wing (χ^2^ = 55.65, df = 1, *P <* 0.001) and in the leg (χ^2^ = 49.35, df = 1, *P <* 0.001).

### p-Chip readability.

Over the course of our experiment, there were zero instances of unreadable p-Chips in the wing (Fig. 6). In contrast, ~23% of p-Chips implanted in the leg were unreadable on PID 1—1 day after implantation—and this doubled to 46% by PID 95 (Fig. 6); however, the effect of Day was not significant (χ^2^ = 0.56, df = 1, *P =* 0.45).

All p-Chips were injected with their photocells facing outward, yet we recorded 67 instances where the orientation of the tag had flipped, as confirmed by obtaining a successful read by scanning the ventral surface of the wing (*n* = 54) or the opposite side of the leg (*n* = 13). In the subset of 13 bats with tags in both the wing and leg, for each animal we counted the number of days, between PID 1 and PID 95 (*n* = 10 days total), with a successful ventral scan for each location. The mean ± SD proportion of days with a successful ventral scan in the wing (0.16 ± 0.28, *n* = 21 flips) and leg (0.10 ± 0.16, *n* = 13 flips) did not differ (*t*_12_ = 0.63, *P* = 0.544, 95% CI [−0.15, 0.28]). In 1 bat that died after our study concluded, we could not read its p-Chip in the leg using any wand orientation, so we dissected the patagium around the tibia and visually confirmed that a “chip flip” had occurred in situ and that the tag was still readable.

### Animal health.

During implantation, we observed instances of bleeding that were promptly stopped with hemostatic powder and/or small ephrin balls. Routine health checks throughout our study found instances of scar tissue buildup around injection sites, but we saw no obvious effects of p-Chip implantation on bat behavior or health. Some bats developed dry skin and/or hair loss, but these changes occur seasonally among bats in the captive colony and thus were not directly associated with tag implantation or animal handling. Our sample size decreased over time because 20 bats died from an unknown cause, mainly from November 2020 to February 2021. To our knowledge, these deaths were not associated with tag implantation or handling because no data were collected during this time, no inflammation was observed at the implantation sites, and bats without p-Chips also succumbed to illness.

## Discussion

Overall, the results of our study suggest that p-Chips are a feasible bat marking technique and that, of the 2 implantation sites we tested, the second metacarpal is preferred due to the relative ease and efficiency of locating and scanning the microtransponder. Below we discuss the rationale for this conclusion in more detail.

### Handling time.

The 2 persons collecting data were experienced bat handlers, with one (RP) having shorter handling times for scanning tags in the wing but not the leg. Handling times remained fairly consistent throughout the study (Fig. 2) but were shorter when scanning p-Chips embedded in the wing versus the leg, likely because it was easier for handlers to open the restrained wing of a bat and expose its metacarpal compared to manipulating and holding its tibia.

### Scan time.

Average scan times increased from PID 1 to PID 95 for p-Chips in both the wing and leg but did not differ between the 2 implantation sites (Fig. 3). Scanning may be hampered by a variety of factors, such as transponder translocation away from the original implantation site and/or photocells of the p-Chip becoming obscured from the wand, for example, by flipping in situ so that they no longer face outward or as a result of connective tissue buildup around the implant as a foreign body.

### p-Chip visibility.

Tags implanted in the leg were less visible compared to those implanted in the wing (Fig. 4), likely because the skin of the uropatagium in *E. fuscus* is darker, thicker, and looser around the tibia. Tag visibility is important; it increases the accuracy of wand positioning and in turn contributes to scanning efficiency. The visibility of subcutaneous tags may be impacted by the deposition of scar tissue at the injection site. Together, these factors may have interfered with the ability of the laser to activate tag photocells, resulting in a higher proportion of unreadable p-Chips implanted in the leg compared to the wing (Fig. 6). This may have further contributed to longer handling times for bats with p-Chips in the leg (Fig. 2).

### Wand flashes.

The number of wand flashes can be used as a proxy measure of unsuccessful reading attempts. The number of wand flashes decreased over the study for the wing and leg implantation sites (Fig. 5). There was also a small difference in the number of wand flashes for a successful p-Chip read between these sites. This latter result was unsurprising given large differences in the proportions of visible tags (Fig. 4) and successful reads (Fig. 6) between the wing and leg. The decrease in number of wand flashes over time likely resulted from increased user experience (i.e., practice positioning the wand and scanning chips).

### p-Chip readability.

The readability of p-Chips differed markedly between the wing and the leg (Fig. 6). All transponders implanted in the wing remained readable, whereas the proportion of readable p-Chips in the leg was lower and more variable over time. This finding is consistent with lower visibility of p-Chips in the leg (Fig. 4). Reorientation of p-Chips at the implantation site is known to influence reading success. For example, in laboratory mice postmortem histology found that p-Chip reorientation renders tags unreadable when the photocells face the tail vertebrae (Gruda et al. 2010). This is in contrast to subcutaneous PIT tags which can change orientation in the animal after implantation but without loss of function; however, readability can still be impacted when PIT tags translocate to an unexpected location and users determine that the tag is lost (Prentice and Park 1984; Gibbons and Andrews 2004). In some bats we attempted to manually flip the orientation of the p-Chip in situ but were unsuccessful. There were other instances when p-Chips appeared to reorient several times within the skin so that the transponder was successfully scanned dorsally, then ventrally, and then again dorsally across sessions. We speculate that tag translocation and/or chip flipping is more frequent in thick, loose skin that allows more room for p-Chip movement (e.g., the uropatagium). To alleviate this, we encourage manufacturers to design microtransponders with omnidirectional reading capabilities (e.g., Mikhailovskaya et al. 2021).

### Animal health.

Handling by humans can stress bats, particularly during trapping or when they are torpid, and adverse effects of handling are associated with the method of tagging (Barclay and Bell 1988; Kunz and Weise 2009). Our bats were from a captive colony, used to regular handling, and typically remained calm during p-Chip implantation and subsequent scanning trials, suggesting that our protocol did not adversely affect them. Some bats bled at the injection site immediately following implantation but this was easily and quickly treated. In a field study of the world’s smallest bat, p-Chips were implanted in 277 Kitti’s Hog-hosed Bat (*Craseonycteris thonglongyai*) with no signs of damage or inflammation in 70 recaptured individuals (Ngamprasertwong et al. 2022). In mice, marking with p-Chips is thought to minimize implantation stress owing to small size of the tag (Gruda et al. 2010).

### Comparison between marking techniques.

Table 2 summarizes and compares the characteristics of split-ring bands, PIT tags, and p-Chips used to mark bats. Relative to conventional forearm bands and PIT tags, p-Chips are much smaller. They also require a smaller diameter injection needle than PIT tags (PIT tag, 12-gauge, outer diameter = 2.769 mm; p-Chip, 21-guage, outer diameter = 0.819 mm; Biomark, Boise, Idaho; https://www.biomark.com), which in turn can be expected to pose less risk to animal health. Because PIT tags require a large injection needle, they are more susceptible to expulsion from the body via the puncture site. By contrast, we noticed only 1 instance where a p-Chip was expelled during implantation. Bats can damage (i.e., make illegible) and/or remove plastic split-ring bands by chewing on them.

**Table 2.** Features of split-ring bands, PIT tags, and p-Chips for marking bats. Information on bands/PIT tags comes from many studies, whereas for p-Chips it is based mainly on this report

Tag characteristic	Split-ring bands	PIT tags	p-Chips
Composition	Plastic, metal	Glass capsule	Semiconductor
Invasive Application?	No (external)	Yes (subcutaneous)	Yes (subcutaneous)
USDA/CCAC Rating	Category B	Category C	Category C
Pain/Duration	Little-to-none/short	Minor/short	Minor/short
Application Tool?	Banding tool/pliers; by hand	sterile needle (12 to 16 G)	Sterile needle (21 G)
Application Injury?	No (unlikely)	Yes (bleeding); internal organ damage, death (rare)	Yes (bleeding); possible limb or tendon damage, infection
Post-Application Morbidity/Mortality	Short- and long-term skin irritation (inflammation or infection); restricted circulation/death (rare)	Inflammation, infection/death (rare)	Possible inflammation, infection/not reported
Affects Behavior?	Yes (bats may scratch or chew band)	Not reported	Not reported
Location	Forearm (typical) thumb or leg (atypical)	Nape/back (between shoulder blades)	No standard location^a^
Code	Analog (engraved on band)	Digital RFID (alphanumeric code)	Digital RFID 9-digit (alphanumeric code)
Size	2 to >6 mm diameter	1 to 4 mm diameter; 8 to 32 mm length	500 × 500 × 100 µm (l × w × h)
Bat Size Restriction?	None	None	Not tested (likely none)
Removable?	Yes (also by the bats)	No (requires surgery)	No (requires surgery)
Reusable?	Yes	Yes (uncommon; requires sterilization)	Not tested; (requires sterilization)
Reader	Visual inspection	Built-in display or wireless connection	Laser wand USB connected to computer)
Reader Range	~0.5 m (by eye)	≤500^b^ mm	≤10 mm
Reader Orientation	Band surface	N/A	Chip surface with photocell
Visible?	Yes (bat must be in hand to read unique number)	No (under skin/fur)	Yes (varies with skin pigmentation)
Persistence	Lifetime (bats can damage by chewing)	Lifetime	Lifetime (but tag can flip and be obscured in situ)
Tag Cost	<$1.00 USD	≤$10.00 USD	$2.00 USD
Reader Cost	N/A	$300 to $2,000 USD	$2,000 USD
Availability/Compatibility	Multiple suppliers/cross compatible	Multiple suppliers/not all cross compatible	p-Chip Corp./internally compatible
Field-Tested?	Yes	Yes	No

^a^Results of present study suggest second metacarpal as a prospective location for small- to medium-sized bats.

^b^Varies with tag and reader model; automated readers can be mounted at roost entrances or on a pole to scan clusters of bats.

Scanners used to read p-Chips and PIT tags differ in notable ways. Critically, PIT tag readers have less stringent proximity and orientation requirements. The p-Chip laser wand that we used (model WA-4000) must be within <8 mm of the implant, whereas PIT tag readers can work at distances of 45 to 500 mm, depending on the model. The hand-held readers for PIT tags and p-Chips also differ in usability. Many different PIT tag scanner models exist, with some portable (e.g., pocket scanners), some stationary (e.g., circular antenna installed at animal entrance/exit points), and others designed to work as arrays to increase the effective reading range. Similar scanner designs may be challenging to incorporate for p-Chips because the photocells are located on 1 surface of the tag and require precise alignment with the reading wand. Furthermore, not every PIT tag can be scanned by every PIT reader because both must function on the same radio frequency to communicate with one another (Gibbons and Andrews 2004). In contrast, the p-Chip technology is proprietary and users rely on a sole source supplier. Use of the p-Chip wand obligates connecting to a computer to record transponder ID numbers, whereas PIT tag readers typically have a built-in display. Lastly, there are significant cost differences between p-Chip and PIT tag technologies (Jolley-Rogers et al. 2012). Although individual p-Chips are less expensive to deploy than PIT tags (Smyth and Nebel 2013), the cost of a p-Chip laser reader wand is higher than most PIT tag readers (Table 2).

The use of any marking method comes with risks. Despite miniaturization leading to low invasiveness, and potentially minimal impact on animal health and well-being, using p-Chips to mark bats poses a set of operational challenges, mostly related to locating and reading the implanted transponder. For example, we know that p-Chips remain visible in the bat wing for at least ~1.3 years, but their permanence beyond this is unknown. Our work in captive bats did not record instances of unreadable p-Chips in the wing (Fig. 6) and ca. 70% of these tags remained visible over time (Fig. 4). The reduced visibility of p-Chips implanted in the leg increased the time to find and scan them (Fig. 3). We noticed instances where a p-Chip in the leg was deemed unreadable one day but gave a viable read on a subsequent day (*n* = 9). While it is possible for p-Chips to be expelled from the body, which would affect estimates of marked versus unmarked individuals, we recorded only 1 instance of tag loss. Retention of p-Chips and PIT tags has been examined and compared in different fish species (Chen et al. 2013; Faggion et al. 2020; Moore and Brewer 2021).

### Other considerations.

Researchers working with tagged insects have obviated the need for handling by placing p-Chips in highly visible and standardized locations (Jolley-Rogers et al. 2012) or by designing housing to guide insects through narrow spaces for efficient wand reading (Robinson et al. 2009; Robinson and Mandecki 2011). Several studies have developed similar approaches for automated PIT tag reading in bats crawling through entrances to roosts or hibernacula (e.g., Silvy et al. 2012; Britzke et al. 2014; Norquay and Willis 2014). For now, using p-Chips for marking bats may be restricted to situations when animals are directly handled.

The ability to distinguish marked and unmarked animals is vital in recapture studies of free-ranging populations. Mark–recapture work requires tags that persist and remain visible/detectable, ideally over the lifespan of an animal. Compared to external tags for marking bats (see Kunz and Weise 2009), p-Chips are highly inconspicuous. This reinforces the importance of standardizing the implantation site when considering the wider adoption of p-Chips to mark bats, as in laboratory mice (Gruda et al. 2010) and fish (Moore and Brewer 2021).

Our results support the conclusion that the relatively translucent, thin, and tighter skin surrounding the second metacarpal of *E. fuscus* is a better p-Chip implantation site compared to the darker (opaque), thicker, and looser skin of the uropatagium around the tibia. But these characteristics will vary in other bat species, depending on their size and morphology. For example, the second metacarpal may be an unfeasible implantation site in bats smaller than *E. fuscus* because the gauge of the needle may exceed the width of the bone and this could tear the chiropatagium. In smaller-bodied bats such as *Craseonycteris*, implanting p-Chips in the forearm may be feasible. On the other hand, in larger bats with robust skin such as *Artibeus*, *Phyllostomus*, or *Cynopterus*, locating and scanning forearm p-Chips may be problematic. Because tail anatomy differs markedly among bat families—in many species the tail moves freely within the uropatagium while in others it is completely lacking, plus some bats have a densely haired uropatagium—this renders the tail as an impractical site for implantation. Ultimately, researchers may have to designate taxon-specific standard sites for implanting p-Chips in Chiroptera.

Despite the above caveats, the feasibility of p-Chips must be field-tested in different species of free-ranging bats, preferably in settings where there is high likelihood of recapturing individuals. For now, we recommend pairing p-Chips with another marking method—such as bands or PIT tags—or marking animals with 2 p-Chip transponders (e.g., 1 in each wing) to aid in the assessment of tag visibility, readability, retention, and localization over time.

While the results of our pilot study are encouraging and warrant further field testing, *we caution researchers against using p-Chips as the sole method for marking bats at this time*, because the consistency of applying this proprietary technology across bat taxa and in different settings remains unknown, which could pose risks to long-term data integrity. Since revising this manuscript our original laser wand (model WA-4000) was recalled by the p-Chip Corp. and replaced with a newer model (WA-6000) to comply with regulations for Class 3R laser products from the Center for Devices and Radiological Health. The new model has reduced laser pulse power, emits fewer pulses during tag reading, and has a smaller spot size, but is reported to activate p-Chips at a longer distance (up to 15 mm). The new model has been tested in mice and fish but not in bats. We conducted a preliminary test with the upgraded wand on 6 thawed *E. fuscus* cadavers from our original study and 2 recently tagged live individuals, and observed variation in scanning performance between 2 operators. The decrease in laser spot size and pulse emissions may reduce the efficiency of scanning subcutaneous p-Chips, especially when the tags are not visible. We suggest that researchers experimentally evaluate the scanning efficiency of the new WA-6000 wand in bats, using an approach similar to ours, before deploying p-Chips in the field.

## Supplementary data

Supplementary data are available at *Journal of Mammalogy* online.

**Supplementary Data SD1.**—Movie illustrating subcutaneous implantation and laser wand reading of a p-Chip injected along the dorsal surface of the second metacarpal in the right wing of a yearling big brown bat (*Eptesicus fuscus*). Also shown are instances of successful laser wand reads accompanied by an audible beep and data output to the computer, and unsuccessful tag reads with no beep or data output.

gyae030_suppl_Supplementary_Datas_SD1

## Data Availability

Contact the corresponding author.
